# Editorial: Socially, culturally and contextually aware robots

**DOI:** 10.3389/frobt.2023.1232215

**Published:** 2023-11-03

**Authors:** Olov Engwall, Juan Pedro Bandera Rubio, Suna Bensch, Kerstin Sophie Haring, Takayuki Kanda, Pedro Núñez, Matthias Rehm, Antonio Sgorbissa

**Affiliations:** ^1^ Division of Speech, Music and Hearing, School of Electrical Engineering and Computer Science, KTH Royal Institute of Technology, Stockholm, Sweden; ^2^ Departemento de Tecnología Electrónica, University of Málaga, Málaga, Spain; ^3^ Department of Computing Science, Umeå University, Umeå, Sweden; ^4^ Robots and Sensors for the Human Well-Being, Ritchie School of Engineering and Computer Science, University of Denver, Denver, United States; ^5^ HRI Lab, Kyoto University, Kyoto, Japan; ^6^ Tecnología de los Computadores y las Comunicaciones Department, University of Extremadura, Badajoz, Spain; ^7^ The Technical Faculty of IT and Design, Aalborg University, Aalborg, Denmark; ^8^ Dipartimento di Informatica, Bioingegneria, Robotica e Ingegneria dei Sistemi, University of Genoa, Genoa, Italy

**Keywords:** social robots, cultural awareness, context awareness, reviews-articles, original research

Socially and Culturally Aware Robotics are emergent areas of research that seek to understand how new generations of robots can become aware of and adequately adapt to their environment, and especially, the people that they are interacting with. As social robots start to spread both to new societies worldwide and to new sectors in societies where they were already present, there is a larger variety in the social context in which they are used, and the robots’ ability to use contextual information of unstructured settings is hence paramount to handle different situations. Moreover, as robots become abundant in societies, it is important that their interaction is inclusive for users of all socio-cultural backgrounds, regardless of personal characteristics such as age, familiarity with technology, linguistic level, cultural background or special needs. This Research Topic of articles contributes to the knowledge of how robots can be made adaptable to different socio-cultural contexts. Theoretical grounding is provided by two different literature reviews of the use of robots to support language learning (van den Berghe, Rohlfing et al.) and summaries of cultural aspects in previous work on backchannels (Engwall et al.) and accents (Obremski et al.). The theoretical perspective is complemented by original research experiments (Tewari and Lindgren, Buyukgoz et al.; Obremski et al.; Engwall et al.) that bring new insights regarding different aspects of socio-cultural interaction with robots. Finally, as a response to the need to adapt to different groups of users, Louie et al. present a framework for participatory design for culturally-aware educational robots and demonstrate how it can be used with children in multilingual settings.

The two reviews of previous work on robot-assisted second language learning by van den Berghe and Rohlfing et al. conclude that the full potentials for educational and social interaction are yet to be fulfilled. van den Berghe finds that robots’ potential to communicate in more than one language is not explored, since most learning interactions were almost exclusively in either the target second language (L2) or the first language (L1) of the student group. She issues a call for more use of systematic translanguage switches in robot-assisted language learning to make robots more aware culturally (supporting students from diverse linguistic backgrounds) and contextually (employing didactic strategies of when the L1 or the L2 is most effective for learning). Rohlfing et al. identify limitations regarding, firstly, the robot’s inability to process multimodal social signals from the learner required to become aware of the context; secondly, that the interaction is often not truly social, since it is focused on an intermediate learning platform, such as a tablet, which restricts the social bonding between learner and robot; and, thirdly, the restricted repertoire of roles that educational robots are given. Starting from the traditional roles of tutor, peer or tutee, they propose additional social roles and specify the necessary perceptual, cognitive and dialogical skills that the robot should possess for each role and different learning activities that the robot could engage in.

Tewari and Lindgren and Buyukgoz et al. focus on strategies to make robots aware of and react to humans (respectively, their emotions and intentions), the human-robot interaction (social norms) and the physical context. Tewari and Lindgren study the specific context of how breakdown in the communication with robots is perceived by different age groups. They show that younger and older users differ in their preferences related in particular to social norms (that were more important to older users) and functional aspects (highlighted more by younger users). Buyukgoz et al. describe a system that makes robots proactive with respect to making prediction about human intention and/or changes in the environment. By testing the system in a domestic robot, they show that more appropriate socio-cultural robot awareness is achieved when combining models of human intention and potential context changes.

Engwall et al. and Obremski et al. address how cultural aspects, of, respectively the humans and the robot, affect the interaction and the humans’ perception of it. Engwall et al. describe how different socio-cultural groups react to socially adaptive robot backchannels. That is, the robot already attempts social awareness by responding in an interaction with two human interlocutors in such a way that their spoken participation should become more balanced. To achieve this, the robot provides more, and more explicit, backchannels towards the interlocutor who has spoken less. Since the human interlocutors have different cultural backgrounds (being L1 or L2 speakers of different gender and age) this may influence how they respond to backchannels from the robot, and the study aims at understanding how the robot should adapt backchannels to different interlocutor groups in order to be culturally aware. Obremski et al. instead investigate how human perception of a virtual social robot depends on whether it has a native or a non-native accent of English. Accent and grammatical errors often influence the perception of, e.g., likeability, trustworthiness and competence in interactive virtual agents and robots, as well as in humans. Previous studies have found that subjects prefer an accent matching their own, as this establishes a shared cultural bond, but contrary to these expectations, Obremski et al. find that German speakers were more negative towards a robot with German-accented English than were native English speakers and that the opposite was true for a native English-speaking robot.

Fittingly, since all above studies clearly demonstrate that the socio-cultural background of the users influence how they perceive robots and that adaptation of the robots to this is a prerequisite for optimal interaction, Louie et al. present a culturally founded framework for involving students, parents and teachers in co-designing student-robot interaction in multicultural settings. The framework consists of three stages, illustrated by three studies, in which the authors have, respectively, interviewed students about their preferences and expectations on educational robots; co-designed the features of an educational robot by letting students provide suggestions on the face, body and interaction of an in-house robot; and tested child-robot interaction in a language learning experiment that permits to observe the children’s responses to the robot.

